# Barriers in the Delivery of Emergency Obstetric and Neonatal Care in Post-Conflict Africa: Qualitative Case Studies of Burundi and Northern Uganda

**DOI:** 10.1371/journal.pone.0139120

**Published:** 2015-09-25

**Authors:** Primus Che Chi, Patience Bulage, Henrik Urdal, Johanne Sundby

**Affiliations:** 1 Peace Research Institute Oslo (PRIO), PO Box 9229, Grønland, Oslo, Norway; 2 Institute of Health and Society, University of Oslo, PO Box 1130, Blindern, Oslo, Norway; 3 International Organization for Migration, Plot 6A, Naguru Crescent, Kampala, Uganda; University of Stirling, UNITED KINGDOM

## Abstract

**Objectives:**

Maternal and neonatal mortality and morbidity rates are particularly grim in conflict, post-conflict and other crisis settings, a situation partly blamed on non-availability and/or poor quality of emergency obstetric and neonatal care (EmONC) services. The aim of this study was to explore the barriers to effective delivery of EmONC services in post-conflict Burundi and Northern Uganda, in order to provide policy makers and other relevant stakeholders context-relevant data on improving the delivery of these lifesaving services.

**Methods:**

This was a qualitative comparative case study that used 42 face-to-face semi-structured in-depth interviews and 4 focus group discussions for data collection. Participants were 32 local health providers and 37 staff of NGOs working in the area of maternal health. Data was analysed using the framework approach.

**Results:**

The availability, quality and distribution of EmONC services were major challenges across the sites. The barriers in the delivery of quality EmONC services were categorised into two major themes; human resources-related challenges, and systemic and institutional failures. While some of the barriers were similar, others were unique to specific sites. The common barriers included shortage of qualified staff; lack of essential installations, supplies and medications; increasing workload, burn-out and turnover; and poor data collection and monitoring systems. Barriers unique to Northern Uganda were demoralised personnel and lack of recognition; poor referral system; inefficient drug supply system; staff absenteeism in rural areas; and poor coordination among key personnel. In Burundi, weak curriculum; poor harmonisation and coordination of training; and inefficient allocation of resources were the unique challenges. To improve the situation across the sites, efforts are ongoing to improve the training and recruitment of more staff; harmonise and strengthen the curriculum and training; increase the number of EmONC facilities; and improve staff supervision, monitoring and support.

**Conclusions:**

Post-conflict health systems face different challenges in the delivery of EmONC services and as such require context-specific interventions to improve the delivery of these services.

## Introduction

Improving maternal and neonatal health is particularly challenging in conflict, post-conflict and other crisis settings [1–6]. This is partly associated with the delivery of disrupted and fragmented health services as health systems in such settings are characterised by damaged infrastructure, limited human resources, weak stewardship and a proliferation of poorly organised non-governmental organisations [7]. Maternal and newborn health in crisis settings is therefore a global problem. The 2011 World Development Report suggested that no low-income conflict-affected country had achieved a single MDG [8] and all were furthest away from achieving any of the MDGs [9]. Although minor improvements have been observed since then, the global outlook of maternal and newborn health in conflict-affected settings remains gloomy. For example, a recent study [5] found that countries that have recently experienced an armed conflict tend to have higher rates of maternal mortality compared to those that have not experienced such conflicts.

With the extremely poor maternal and newborn health outcomes in crisis settings, a critical intervention to improve the situation has been ensuring the availability and utilisation of quality emergency obstetric and neonatal care (EmONC) services. These are services necessary to save life and are most useful when a complication occurs during pregnancy, childbirth and after birth. With one in five women of childbearing age likely to be pregnant in any crisis setting [10], coupled with an estimated 15% of pregnant women in developing countries expected to experience pregnancy-related complications [11], and with neonatal deaths accounting for over 40% of all deaths in children younger than five years of age [2,3], the importance of EmONC services to women and neonates in such settings cannot be over emphasized. Since 1995 the Inter-agency Working Group (IAWG) on Reproductive Health in Crises (a broad-based, highly collaborative coalition of 18 Steering Committee member agencies–representing UN, government, non-governmental, research, and donor organizations) has been at the forefront of expanding and strengthening access to quality sexual and reproductive health services for people affected by armed conflicts and natural disasters. They have also developed ‘a coordinated set of priority activities designed to prevent and manage the consequences of sexual violence; reduce HIV transmission; prevent excess maternal and newborn morbidity and mortality; and plan for comprehensive reproductive health services’ [12] during crisis situations, known as the Minimum Initial Service Package (MISP) for Reproductive Health in Crises. One of the priority activities proposed in the MISP to prevent excess neonatal and maternal morbidity and mortality is to initiate the establishment of a referral system to manage obstetric emergencies [13]. The IAWG equally advocates that once the acute stage of an emergency has passed and the emergency moves into the post-conflict/recovery phase comprehensive reproductive health services including EmONC must be implemented.

Based on the range of EmONC services that a facility provides it can be classified as a basic EmONC (BEmONC) or a comprehensive EmONC (CEmONC) facility. In this regard, CEmONC services are generally expected to be provided at hospitals while BEmOC services should be provided at health centres or clinics. To achieve the objectives of saving lives and preventing disabilities, EmONC services must be supported with evidence-based policies, trained health professionals, and efficient referral procedures [14]. In spite of the strong evidence of the importance of EmONC services for improving maternal and newborn health, access to quality emergency obstetric care services in conflict and post-conflict settings is a recurring challenge [15–18]. Additionally, a recent survey of current practices and programmes for improving neonatal survival in humanitarian settings among key humanitarian actors identified a number of challenges including lack of funds, gaps in training, and staff shortages and turnover [2]. In post-conflict Iraq, another study [19] revealed that only about a quarter of hospitals in 8 of the 18 Governorates could offer emergency obstetric care services. The effective delivery of such services was hampered by falling standards of training and regulation; lack of drugs, supplies and equipment; inadequate staff due to external migration and premature death; high levels of insecurity of patients referred or admitted to hospitals among others [19].

The current UN guidelines recommend at least four BEmOC facilities and one CEmOC facility for every 500,000 people in the population [20]. A global survey on the availability of EmOC services found that CEmOC facilities are largely available to meet this recommended minimum number while BEmOC facilities were consistently insufficient in numbers both in countries with high and moderate levels of maternal mortality [21]. A 2005 need assessment of EmOC services in Uganda found that there was an urgent need to improve the availability of EmOC services as a high number of facilities lacked one or more signal functions [22]. In two recent assessments of the reproductive health situation, including EmOC, in Northern Uganda [23], one of the major challenges identified was gaps in the telecommunications systems to coordinate ambulances and lack of funds for fuel and vehicle repairs. A national EmOC assessment was undertaken in 2010 by the Burundian Ministry of Health in partnership with the WHO, UNICEF, UNFPA and Averting Maternal Death and Disability (AMDD) Program [24] but the findings have not yet been published.

Burundi and Northern Uganda are both recovering from brutal civil wars that claimed tens of thousands of lives and caused millions of people displaced [25–27]. Burundi has experienced cyclical ethnic violence between the Hutu and Tutsi ethnic groups since 1962 following the country’s independence from Belgium [28]. The most recent spell of civil war lasted from 1993 to 2005. However, the country has continued to experience sporadic and isolated incidents of armed violence since the formal end of the conflict. The armed conflict in Northern Uganda on the other hand started as an insurgency launched by the rebel Lord’s Resistance Army against the Ugandan national army, Uganda Peoples Defence Force [29]. The war lasted from 1986 until 2006 and was described by Jan Egeland, the then UN Special Representative for Humanitarian Affairs, as ‘the worst forgotten humanitarian crisis in the world’ [30]. Although the conflict appears to have been rooted in mistrust and perceived marginalization of the Northern region by the government [31], it did not have a strong ethnic outlook. In Burundi the female life expectancy at birth for 2012 stood at 57 years, with a maternal mortality ratio (MMR) of 740 per 100 000 live births and total fertility rate of 6.2 (WHO Global Health Observatory Data Repository), one of the highest in the world. Northern Uganda on the other hand has a total fertility rate of 6.3 (the highest in the country), with a median age at first birth of 17.8 years [32]. The latest Ugandan MMR estimate from the UN indicator monitoring database is 360 per 100 000 live births, and it is likely that the MMR of the Northern region is above the national average. The neonatal mortality rate for Burundi and Uganda are 36 and 23 per 1000 live births respectively [33].

Existing studies on the status of the provision of EmONC services in conflict and post-conflict settings have mainly been quantitative in nature, largely assessing whether or not the basic standards are in place. Qualitative studies exploring the challenges that key EmONC stakeholders (health providers and policy makers) undergo on a daily basis to make these services available for women and newborns and the strategies they adopt to mitigate such challenges are relatively uncommon. This study seeks to contribute to the broader literature on the state of EmONC in conflict and post-conflict settings, focusing on the barriers that frontline health providers and policy makers encounter in the delivery of these important lifesaving interventions. We also seek to highlight some strategies they have put in place to improve the delivery of quality EmONC services. Such contextual information can help policy makers to better design and deliver EmONC services.

This study therefore aims to explore an in-depth understanding of the state of EmONC services in Burundi and Northern Uganda, especially the barriers affecting the efficient supply and delivery of EmONC services as well as existing local strategies to improve services. Our choice of the study sites is based on the variation in the nature and length of the armed conflicts and the similar duration since the conflict ended. The recent conflict is Burundi had a strong ethnic character and lasted for about 12 years, while the conflict in Northern Uganda lasted for about 20 years and was not organised along ethnic lines. Additionally, at the time the fieldwork was conducted it had been about 7–8 years since the conflicts ended. This allows us to compare the challenges in the delivery of EmONC services several years after the formal end of hostilities and the initiatives underway to address the challenges. Furthermore, our choice of research participants is guided by the key stakeholders involved in the provision of EmONC services; frontline clinical staff (healthcare providers), local health administrators (local policy and decision makers), and technical and material support organisations. With such diversity in study sites and research participants, and yet similar post-conflict duration, a more comprehensive outlook of the barriers and strategies in place will be captured. Our main research questions are: ‘*what are the barriers to the effective delivery of EmONC services*?’ and ‘*What are the existing or planned strategies to improve the delivery of EmONC services*?’ We shall identify the contextual factors that interplay to affect the effective delivery of these services. The findings will provide context-specific evidence to local EmONC policy makers to improve the delivery of EmONC services in their respective countries.

## Materials and Methods

### Ethics Statement

Ethics approval for the study was obtained from the Regional Committee for Medical and Health Research Ethics, South-East (Norway); le Comité National d’Ethique pour la Protection des êtres Humains Participant à la Recherche Biomédicale et Comportementale (Burundi); and Gulu University Institutional Review Committee (Uganda). We also received permission from local administrative and health authorities. All participants provided a written informed consent before participating in the study and their anonymity, privacy and confidentiality was respected.

### Study Sites

The study was conducted from June to September 2013 in three provinces in Burundi and a district in Northern Uganda. The highest administrative unit in Burundi is the province; with each province having a number of communes. On the other hand, Uganda is divided into four administrative regions; Central, Western, Eastern, and Northern, with the regions in turn divided into districts. In terms of size and population, a district in Uganda is similar to a province in Burundi. That is why we choose the second level administrative unit for our study site in Northern Uganda (district) and a first level administrative unit for our study site of Burundi (province). In Burundi the study was undertaken in the provinces of Bujumbura-Mairie, Bujumbura-Rural and Ngozi while in Northern Uganda our study site was the district of Gulu. The Gulu district is made up of 3 counties, 16 sub-counties, 70 parishes and 279 villages, with a population of 374,700 [34]. The 2008 census in Burundi [35] puts the population of the three provinces of Bujumbura-Mairie, Bujumbura-Rural and Ngozi at 497,166, 555,933 and 660,717 respectively.

### Study Participants

Study participants were recruited from among staff of non-governmental organizations (NGOs) and local health providers (LHPs) and only those knowledgeable of or experienced with EmONC-related activities were included in the study. These included frontline healthcare providers at health facilities; senior health administrators and decision makers; organisations involved in the provision of EmONC training, donation, and supply of essential EmONC medicines, equipment and other supplies; and organisations providing other forms of EmONC-related technical and material support within our study areas. The NGOs included local, national and international organizations working in the domain of maternal health, be it at the level of policy support or technical assistance, health system support and strengthening, or delivery of health services. We classified the NGOs into three main groups: NGO-Health providers (NGOs that also provide health services), NGO-Policy makers (mainly UN-based NGOs) and NGOs (non-UN-based NGOs that do not provide health services). The LHPs were drawn from clinics, health centres and hospitals, and included nurses, midwives and doctors working on maternal health issues in their institutions, mainly at the maternity, antenatal care, and obstetric and gynecological units in both public and private facilities. Others included senior administrators at ministries of health at the provincial, regional or district levels (LHP-Policy makers).

### Data Collection Methods

This is a qualitative case study that used face-to-face semi-structured in-depth interviews (IDIs) and focus group discussions (FGDs) for data collection. Interviews and FGDs were conducted in the local language, French or English (where applicable) by the principal investigator (PCC) or trained local research assistants (RAs). All interviews and FGDs were guided by detailed ‘Interview and FGD guides’ that were developed in both the English and the French languages and piloted prior to the commencement of study. The complete ‘Interview and FGD guides’ have been reported elsewhere [36].

### Conducting Interviews and FGDs

Interviews and FGDs with NGO staff and local health providers were held mainly at their places of work, and the lawn of some local hotels. All interviews in French and the local languages were undertaken by the trained local RAs while all the English interviews were undertaken by the principal investigator (PCC). Interviews and FGDs typically lasted from 50–130 minutes. The FGDs included between 5–8 participants. Interviews and FGDs were audio-recorded and field notes taken. Soft drinks, tea or coffee was provided to FGD participants during the discussion. We also provided transport reimbursement to FGD participants.

A total of 42 IDIs and 4 FGDs were conducted across the study sites as shown on Table 1. This involved a total of 69 participants; 30 males and 39 females. In terms of their participant category, 32 were LHPs and 37 NGO staff.

**Table 1:** Number of interviews and FGDs, by study site and participant category.

		Participants		Total
Country	Study areas	Local Health Providers	NGOs	
Burundi	Bujumbura Marie, Bujumbura Rurale, and Ngozi provinces	9 Interviews & 1 FGD	11 Interviews & 1 FGD	20 interviews & 2 FGDs
Uganda	Gulu district	12 Interviews & 1 FGD	10 Interviews & 1 FGD	22 interviews & 2 FGDs
All countries	All study areas	21 interviews & 2 FGDs	21 interviews and 2 FGDs	42 interviews & 4 FGDs

### Research Assistants

In each of the settings, RAs were recruited locally. A total of 6 RAs were recruited and trained across the study sites; 4 in Burundi and 2 in Northern Uganda. All RAs understood the local language(s) plus English and/or French and were educated up to the university level. In Burundi interviews and FGDs were mainly held in the French or Kirundi languages, while in Northern Uganda they were held in English. The RAs were also involved in the transcription of the audio files and translation of the transcripts into English (where applicable).

### Data Management and Analysis

All interviews and FGDs were transcribed and translated into English (where applicable). The English transcripts were then imported into the QRS Nvivo software (QSR International, 2012). The data was analysed using the framework method [37]. Three team members (including PCC and PB) open-coded the transcripts on Nvivo and Microsoft® Word (where the texts of interest are highlighted and the code first labelled using the ‘*New Commen*t’ sub-menu under the ‘*Review*’ menu). Microsoft® Word was used for coding and analysis by one of the co-authors who did not have access to Nvivo. The codes were mainly descriptions and labels of specific concepts as the transcripts were read. Two team members reviewed the codes that were developed and the inter-coder reliability was high. Related codes were then collapsed into different categories, and the categories were subsequently grouped into specific themes. The themes were inductively and deductively developed, involving predetermined themes included in the interview and FGD guides and explicitly covered during the data collection and review, as well as those that emerged during the data review. There was therefore a constant interplay between data collection, analysis and theme development, with dominant themes that emerged in earlier interviews and FGDs being explored deeper in subsequent and later interviews and discussions. The theme development was jointly undertaken by three team members.

## Results

In the paragraphs that follow, we present the participants’ perspective on the state of EmONC services during the conflict; current state of EmONC services; and barriers to the delivery of EmONC services and existing strategies to address the challenges to delivery in Burundi and Northern Uganda.

### EmONC services during the conflict

The state of EmONC services during the conflict across the study sites was largely perceived as deplorable. According to the respondents, the conflict affected EmONC services in two major ways. The first was the lack of basic EmONC medications and equipment in many health facilities, depriving many women and newborns of basic lifesaving services. Another major issue was the breakdown of the referral system associated the high level of insecurity at the time, characterised by numerous road blocks, travel restrictions between lower level health facilities in rural areas and specialised EmONC facilities in the urban areas, poor communication between health facilities, and fleeing of essential personnel. These resulted in preventing the transfer of women with severe complications to referral facilities where care is provided; and acute shortage of skilled EmONC personnel to provide basic services.

### Current state of EmONC

The main issues raised with respect to the current state of EmONC services were focused around accessibility, availability, quality, and geographical distribution.

#### Burundi

In Burundi, participants’ perceptions on the availability and quality of EmONC services were mixed. A number of policy makers in MRH felt that poor availability and quality of EmONC services was one of the most pressing health challenges facing Burundi. One of the policy makers working with an international NGO pointed to the fact that a national EmONC assessment undertaken in 2010 found that less than 2% of all the health facilities in the country offered the required standard EmONC functions (basic and comprehensive).

“*The basic and comprehensive EmONC in health facilities in 2010 was 1*.*8%*. *It wasn’t enough…At 1*.*8%*, *they don’t exist*!” NGO-Policy maker, IDI–Bujumbura

Other respondents acknowledged the poor state of EmONC services in Burundi in 2010, but however emphasised that some important improvements have taken place since the last national assessment was undertaken. These included among others the building of new health facilities and the installation of some EmONC functions.

“*In 2010*, *there were some hospitals newly built which did not carry out caesarean section and blood transfusion*. *Since 2011*, *they began to provide such services*. *Today the number of those facilities has increased*.” NGO, FGD–Bujumbura

One major quality concern raised by the most of the respondents was the poor development of neonatal care signal functions across many health facilities.

*“On maternal health*, *services are found in every health facility*, *the issue is at the level of neonatal care*. *This kind of treatment is not found everywhere”* NGO, FGD–Bujumbura

Concerning the accessibility and geographical distribution of EmONC services, most participants reported that the number of CEmONC facilities and specialists were very few and located mainly in urban areas, creating a huge equity gap in access to CEmONC services between urban and rural areas.

“*In the country*, *the CEmONC structures are still few*. *We have 66 whereas we are supposed to have 163…*” NGO-Health provider, IDI–Bujumbura

“…*all the specialists are concentrated in town*. *Even if they were a lot*, *they are concentrated in one region*, *and it is a problem*” NGO, FGD–Bujumbura

#### Northern Uganda

An overwhelming number of participants in Northern Uganda were very critical about the availability and quality of EmONC services in the area. Attention was drawn to the fact that the most basic of EmONC supplies such as a blood pressure machine or uterotonic drugs were unavailable in some EmONC-designated facilities. A few respondents however felt that with the construction and equipping of more facilities in rural areas coupled with the recruitment of qualified personnel, the situation has been improving.

“…*I would say efforts have been made for instance by WHO* (World Health Organization) *in terms of building their capacities*. *They have trained the core health workers in the districts*, *that is*, *the midwives and then the clinical people in terms of managing the emergency obstetric care*. *They have even gone ahead to provide them the equipment*.*”* NGO-Policy maker, IDI–Gulu

When prompted on the coverage of EmONC services in Gulu, most participants were however uncertain as an up-to-date mapping of the status of availability and quality of EmONC services has not been undertaken in the district of Gulu. Most of the respondents reported that although in principle all hospitals should be providing CEmONC services while all health centres provide BEmONC services, that this was not the case. For example, higher level health centres (Health Centre IV) in Northern Uganda that are expected to provide CEmONC services are unable to do so because the theatres were poorly designed when the facilities were constructed, making them unable to undertake caesarean sections.

With respect to the geographical distribution of EmONC facilities, BEmONC services were generally perceived to be more available and accessibility to the general population compared to the CEmONC services. Most respondents felt that while BEmONC facilities have increased in number over the past years, the same cannot be said of CEmONC facilities.

“*Basic emergency obstetric care is not bad because all health centres IIIs upwards and some health centre IIs actually have functional delivery units*, *they have the oxytocin*, *parenteral antibiotics*, *anticonvulsants and many of them have at least one midwife who is trained in early newborn care and management of the postnatal period*” NGO, IDI–Gulu

Among the participants, there was unanimity that the neonatal components of EmONC were still seriously under-developed and in many cases neonates who need these services are at risk of dying or ending up with serious disabilities. In fact, in one of the main EmONC referral facilities in Gulu district, the neonatal unit was actually non-functional at the time of the study.

### Barriers to effective delivery of EmONC services

From the analysis of the interviews and FGDs across the study sites, two major themes and 16 sub-themes emerged as the barriers to effective delivery of quality EmONC services. The two major themes are human resources-based challenges, and systemic and institutional failures. A summary of the themes and sub-themes are presented in Table 2. Of the 16 barriers reported, 7 were common to both Burundi and Northern Uganda, 6 were common only in Northern Uganda, and 3 were common only in Burundi. In the paragraphs that follow we describe the barriers identified with respect to the study sites.

**Table 2:** Major themes and subthemes related to perceived barriers to the delivery of quality EmONC services in Burundi and Northern Uganda.

		Study settings	
Themes	Subthemes	Burundi	Northern Uganda
**Human resources-related challenges**			
	Acute shortage of trained personnel	X	X
	Demoralised personnel and perceived lack of recognition		X
	Perceived poor living conditions and poor remuneration for personnel	X	X
	High personnel turnover	X	X
	Increasing workload and high burn-out	X	X
	High levels of staff absenteeism in rural health centres		X
	Poor level of coordination among key EmONC personnel resulting in delays to provide emergency services		X
**Systemic and institutional failures**			
	Poorly operational ambulance service for referrals		X
	Inefficient drug supply system		X
	Inefficient referral system		X
	Lack of essential installations, supplies and medications	X	X
	Poor allocation of limited resources	X	
	Poor harmonization and coordination of EmONC training curriculum nationally	X	
	Weak/ incomprehensive training curriculum	X	
	Poor data collection and monitoring system	X	X
	Inequity in the distribution of EmONC facilities between urban and rural areas	X	X

EmONC: Emergency Obstetric and Neonatal Care.

### Burundi

#### Human resources-related challenges, Acute shortage of trained personnel

Participants were unanimous that the EmONC workforce was inadequate, especially in the rural areas. Many attributed this partly to the period of the conflict when many health personnel fled out of the community for their personal safety. Other participants also associated the shortage of EmONC personnel to the introduction of the universal healthcare policy for pregnant women and under-five children that has led to an increase in the demand for EmONC services. Many respondents felt that the increase in the demand for maternal, reproductive and child health services have not be complemented with a corresponding increase in the workforce. The major cadres of EmONC personnel in extreme shortage were midwives and medical doctors.

“*The problem nowadays is that there are fewer health personnel at the health centre with so many patients*. *It even affects the quality of the services provided*. *For instance*, *two nurses may consult about 60 patients a day while one is supposed to consult about 15 in order to perform efficiently*.” NGO, IDI–Ngozi

Related to the barrier of acute shortage of health personnel was the high turnover of health personnel. A number of NGO respondents that provide EmONC-related training in the country lamented the fact that plenty of resources are devoted into the training of health personnel on EmONC but many of these trained personnel tend to move to other services, creating a perpetual shortage of EmONC-trained personnel in EmONC-designated facilities. This high turnover was also blamed for the overall shortage of EmONC personnel in the country.

“*You train someone today in one service and tomorrow he is working in another service*. *This affects the utilisation and quality of these services as the new beneficiaries may come and don’t find the service…So*, *we keep on training new health workers…*” NGO-Policy maker, IDI—Bujumbura

Additionally, some participants felt that the country has always been struggling with the problem of shortage of health workers but since the introduction of the universal healthcare policy for pregnant women and under-five children, the workload has steadily been increasing and high levels of burn-out has been observed among health personnel. Some respondents reported that a number of facilities have reduced the number of clients they can attain to because their staff are overwhelmed and burn-out.

“*Our receiving capacity is limited because we have a limited number of qualified staff*…” LHP, IDI–Ngozi

#### Perceived poor living and working conditions

Some participants also felt that their working and living conditions are undermining their ability to deliver quality EmONC services. This was particularly the case with public sector personnel living and working in the rural areas. Many made mention of poor salaries that were not adequate to meet their needs as well as lack of some essential EmONC supplies as the main areas of concern.

#### Systemic and institutional failures, Poor allocation of resources

Many respondents, especially among the international NGOs felt that the pattern in the allocation of EmONC-related resources was poorly planned, and has led to inequality in availability and quality of services. They made mention of situations where small health centres with lower number of births and very few qualified staff have been equipped with EmONC supplies neglecting other facilities that have a relatively higher number of births and more skilled personnel. Some key stakeholders found this mode of operation worrying and advocated for a more need-based approach in the allocation of limited EmONC material resources.

“*Equipment have been given but not to the facilities that need them most*. *For example you have a health centre with just one nurse*, *so why do you want to invest in such a facility…so it is important to first identify the right facilities*.” NGO-Policy maker, IDI–Bujumbura

A number of frontline EmONC staff also reported that they occasionally experience the lack of essential EmONC medication and supplies. This was mostly observed when they receive an unexpectedly high number of clients.

“…*there is shortage of materials at times especially when we receive many cases…It may happen that we receive many cases during the day and night*. *The next case may find us without any prepared materials*.” LHP, IDI—Ngozi

Some respondents were of the view that the poor allocation of limited EmONC resources is also a contributing factor to the lack of essential EmONC supplies and medication experienced by some facilities. Additionally, they felt that the increasing volume of clients taking advantage of the universal healthcare policy has not been matched with a corresponding increase in essential supplies.

Furthermore, participants reported that the unequal distribution of EmONC-designated facilities between urban and rural areas adversely affects the delivery of quality EmONC services in rural areas where majority of Burundians live. For example in the Bujumbura Mairie province, the four public CEmONC facilities were all located within Bujumbura city. Another implication for the poor distribution of EmONC facilities was that personnel in the cities tend to be overwhelmed with clients.

#### Poor coordination of EmONC

Some respondents felt the current system of EmONC training in Burundi is not appropriately harmonised and coordinated, with different training institutions and organisations offering different types of training. This means that the effectiveness of the various training programmes and the competence of the trainees might vary across various locations.

Additionally, some participants highlighted important lapses in some of the EmONC curricula currently provided across the country. For instance, some respondents felt that many EmONC training programmes lack a practical component where trainees are able to ‘try-out’ the skills they have learned on training materials. They felt that most training that has been provided in the past has largely been theoretical in nature with very little or no room for practical exercises.

“*Some trainings have been done but it is not effective; it has been theoretical training for EmONC…EmONC cannot be theoretical*, *they have to do practical exercises*.” NGO-Policy maker, IDI–Bujumbura

#### Poor data collection and monitoring system

Participants, especially the policy makers felt that no reliable EmONC data collection and monitoring system exist in the country. Some respondents acknowledged that a national EmONC need assessment was undertaken a couple of years ago although uncertainty lingers around the current status of EmONC services in the country. They were of the opinion that an effective data collection and monitoring system should capture the regular EmONC availability and coverage indicators in addition to information on the effectiveness of the EmONC training programmes.

### Northern Uganda

#### Human resources-related challenges, Shortage of trained personnel and demotivated personnel

Acute shortage of EmONC-trained personnel was a deficiency reported by most of the respondents. This meant that many facilities lacked the necessary manpower to effectively provide quality EmONC services. While it was much easier to recruit nursing assistants, nurses and clinical officers, the recruitment of midwives, general practitioners and gynaecologists was reportedly much harder. This situation was more precarious among facilities in rural settings.

Additionally, many respondents felt that the challenging working environment contributed to demoralise staff, especially those within the public sector. Many local health providers reported that repeated stock-out of essential supplies including medication demotivates and demoralises many personnel as this seriously affects the quality of the services provided.

“…*sometimes you find that you go to the district medical store and they tell you ‘this is not in the store’ and the next ordering day you go ‘this is not there’*. *That one also demotivates somebody because you feel you want to give the services but there is nothing for you to use*.” LHP, IDI—Gulu

Some personnel, especially the midwives also felt a deep sense of lack of recognition, support and motivation within the health system. A common concern raised was the lack of promotion for those that have returned from further training, a phenomenon that further demoralises many.

Furthermore, many participants, especially the policy makers and administrators/supervisors felt that the shortage of EmONC-trained personnel was further exacerbated by a high level of turnover among the personnel. They lamented the fact that new recruits do not stay long on their jobs particularly in the rural areas. Also, some personnel that have been provided specialised EmONC-related training have abandoned their posts and moved elsewhere.

Another major human resource-related challenge raised by the participants was an ever increasing workload, resulting in high burn-out among the personnel. Some of the contributing factors were the high turnover and high levels of absenteeism especially in rural health facilities. In many cases staff had to undertake more tasks and attend to more patients than they normally do. In facilities with only one midwife, she may have to work all year round without a period of leave.

“…*staffing*, *that is the major problem we are facing*!…*so you find one midwife handling antenatal clinic*, *deliveries*, *the EID* (Early Infant Diagnosis) *room*, *the ART clinic*, *PMTCT and emergency [*during the day*]*, *and then at night the same person*, *which is a major problem*.” LHP, IDI–Gulu

#### Poor living and working conditions

Complaints about poor living conditions and poor remuneration were a recurring issue especially among the health personnel in the public sector and those working with religious organisations and in facilities in rural areas. The most challenging concern was that decent and affordable accommodation was difficult to find and this has even discouraged some personnel from staying in the area for a long time. Most of the personnel also felt that the salaries are not catching up with the fast-growing cost of living. Reference was made to a neighbouring country, like South Sudan, where colleagues who went there are better remunerated for their services.

“…*when you see the least salary a nurse earns; 500*,*000 UGX*, *that one in Uganda is nothing*. *But when a nurse gets another job outside*, *say may be in South Sudan or other places where they are paid like 700*,*000 to 900*,*000 plus*, *that one can cater for your children in school and the rest for you*.” LHP, IDI—Gulu

A related challenge raised by many participants was the high level of staff absenteeism in rural health facilities, which was having a severe effect on the delivery of quality EmONC services in those areas. This high level of absenteeism has been partially associated to the very poor living conditions in some rural areas where basic amenities like electricity and good schools for the children of the health personnel are lacking. The result has been that many personnel recruited to work in those areas prefer to stay in town and commute to the workplaces. Another explanation for the high absenteeism is that most of the key personnel spend a lot of time attending meetings, workshops and other events away from their work places where they could make extra money from per diems. Others even have parallel work that they do alongside their government post and as such have to split their time between these jobs.

“…*one big issue […] is the commitment of those who are employed at the health centres*. *In the health unit*, *you might be using a staffing level of about 80%*, *meaning that out of the 15 health workers that are supposed to be there*, *you have 12 or 13*, *which is not so bad*. *But out of those at any one time*, *it is very difficult to find 10 at the health unit*” NGO, IDI–Gulu

#### Poor level of coordination among key EmONC personnel

A number of local health providers lamented on the poor level of coordination among the key personnel involved in the provision of EmONC services, resulting in delays in providing these important lifesaving services. The major recurrent concern raised was difficulties in assembling essential personnel such as the anaesthetist, laboratory technician, theatre nurse, midwife, and surgeon among others, when an emergency caesarean section has to be performed. The situation is particularly precarious at night when some personnel who live far away from the hospital have to be transported to the hospital before the team can perform the task. A number of respondents narrated incidents where caesarean sections have been delayed because some essential personnel could not make it to the hospital on time.

#### Systemic and institutional failures, Inefficient referral system

Participants were generally concerned about the effectively of the ambulance service, especially in assisting with the transportation of emergencies from lower health facilities to the main referral facilities. Poor management and maintenance of the ambulance was the most common concern. In many occasions the patients may have to pay for the fuel in order to be transported as it is regularly out of fuel.

Furthermore, participants felt that the linkage between the referral facilities and lower level referring facilities in terms of the road network, the communication and referral guidelines were inefficient. These challenges are further compounded in some areas by a poor road network, especially the roads linking the rural villages to the main CEmONC facilities in the cities. Also, some were critical that referral guidelines are only on paper as actual implementation remains very weak. Another major concern raised about the referral system was the lack of counter-referrals or feedback between the referring and the referral facilities. As such, there is no tracking on the effectiveness or success rate of the referrals.

“…*when referrals are made no one bothers to follow up if the referrals were effective*. *That also makes the referrals ineffective*. *So who brings feedback that the client who was referred was served*, *what is your success rate of referrals*?” NGO, IDI–Gulu

#### Inefficient drug and related supplies system

Participants also felt that the current drug supply system severely undermines the working conditions and the quality of services offered. The current system is described as the ‘*push system*’, where the decision on which drugs to send to a facility is determined at the central level rather than at the level of the facility concerned. The result has been that some essential supplies are sent in small quantities, which quickly runs out, affecting the quality of services. In some situations public health facilities need to procure the drugs they need in order to provide effective services. This unfortunately comes at a cost to the clients which would not have been the case if the national medical store supplied the local facility pharmacy based on the needs of the facility.

“*They may send drugs that you don’t need and don’t send enough drugs that you instead need…you have to procure drugs on your own at times which is not easy and many women may not be able to afford them*.” NGO-Health provider, IDI–Gulu

Additionally, among the facilities that are designated to provide EmONC services, a good number are still unable to deliver these services. Participants largely associated this to the poor design of some of the facilities that does not allow the provision of some specific signal functions and regular stock-out of some essential EmONC supplies and medications such as antibiotics, uretonics and urinary catheters among others. The current ‘push system’ for the supply of medical supplies was also blamed for the regular lack of essential medicines and supplies.

“*Not all Health Centre IVs have a functional theatre*, *which should be the case and but at least they are more that are functional now and even fewer have blood transfusion services*” NGO, IDI—Gulu

Furthermore, most respondents also reported that there was a huge geographical inequality in the supply, access and quality of EmONC services between urban and rural areas in favour of urban areas. This means that many women in rural areas have to travel over long distances to seek these services, especially CEmONC services that are exclusively located in the urban centres. For example in Gulu, of the three hospitals identified that purported offered CEmONC services all were located in Gulu town.

#### Poor data collection and monitoring system

Participants were generally unaware if any recent EmONC need assessment had been undertaken in Northern Uganda. As such, reliable and up-to-date data on the availability and quality of EmONC services has been poor or non-existent.

### Strategies to improve the delivery of effective EmONC services in Burundi and Northern Uganda

A number of strategies to address the human resources-related and systemic and institutional challenges presented in the preceding section were identified. The major subthemes that emerged across the study sites are as follows.

#### Burundi

The subthemes that emerged from the interviews and FGDs included training and re-training of key EmONC providers, harmonisation and strengthening of the training curriculum, and improving the referral system.

#### Addressing the human resources-related challenges, Training and retraining of essential EmONC personnel

Participants highlighted that some initiatives are ongoing to improve the number of trained EmONC personnel around the country. The main strategy was the training and re-training of health personnel on EmONC functions, and provision of essential supplies to facilities that have received training. The main players involved in this in-service training and material support in Burundi were NGOs such as MSF (Médecins Sans Frontières) and Pathfinder International.

“*In our region for example*, *there is training for Emergency Obstetric and Neonatal Care*. *We have also intervened in giving equipment to health facilities*. *There has been training on how to deal with a newborn who presents with complications and we have established neonatal services in every hospital*. *We have trained health personnel in the prevention of post-partum hemorrhage*.” NGO, IDI–Bujumbura

#### Addressing the systemic and institutional failures, Strengthening EmONC training and referral system

Participants reported that there is an effort initiated by UNFPA to harmonise and strengthen the EmONC training curriculum at the national level to ensure that all institutions involved in training adopt the same curriculum. The new curriculum and training is expected to incorporate a strong practical component.

Additionally, participants highlighted the implementation of some initiatives to improve the overall referral system. For example, NGOs like MSF have a number of vehicles that serve as ambulances in the areas in which they operate. Also, a system of community financing of the ambulance service has been instituted whereby each family living in a particular health district makes an annual contribution towards the running of the public ambulance service. The programme has partially been rolled out in some parts of the country although some respondents felt that it has not been properly coordinated and the collection of the financial contribution from the households has not been consistent. In spite of the criticisms, a number of respondents felt that if it is well managed and coordinated it will be a step in the right direction.

#### Northern Uganda

The major subthemes were more extensive and included the following: training and recruitment of key EmONC personnel; overhauling of the entire referral system; increasing the number of EmONC facilities in rural areas; better personnel support, monitoring and supervision; improving supply of essential materials and medications to facilities; and improving the working conditions and salary situation of staff.

#### Addressing the human resources-related challenges, Supporting the training of EmONC personnel

Some participants reported that some NGOs have responded to the acute shortage of trained personnel with supporting the training of essential EmONC personnel. A case in point was UNFPA-Gulu office that is providing scholarships to government identified candidates to undertake a training course in midwifery with a guarantee of government employment upon completion. The World Health Organization sub-office in Gulu was also identified as a key player in this domain, also providing essential EmONC supplies to some health facilities.

#### Improving working and living conditions for personnel in rural areas

In an attempt to improving the retention and absenteeism rates for health personnel in rural areas in Northern Uganda, some respondents highlighted that regular support, monitoring and supervision of personnel in those areas are being undertaken. This is a concerted effort between the host communities (through the health unit management committees) and the district health office. Some respondents however felt that a major challenge with this strategy has been the inactivity of the health unit management committees in many areas.

Participants also reported that there is a current government strategy to reduce the personnel turnover rate, and attracting and retaining more personnel especially in higher level health centres like health centre IVs and rural areas. The plan is to increase the salaries of those personnel, and the strategy appears to specifically target the medical doctors that are mostly affected by this phenomenon. As health centres IVs are expected to provide CEmONC services, a rehabilitation of the theatres is also expected.

“…*government has decided to increase the salaries of staff*, *of doctors who are working there* (Health Centre IVs) *and to reactivate these theatres because these theatres would be nearer to the population…*” NGO, IDI–Gulu, Uganda

#### Addressing the systemic and institutional failures, Strengthening the referral system

Many participants felt the entire referral system needed an overhaul, especially in addressing the issues of untimely referrals and poorly operational ambulance service. In this regard, several NGOs have been instrumental in providing referral support. For example, UNFPA has been a key player in providing equipment, ambulances and other EmONC related resources to facilitate the timely referral of patients. Other NGOs have equally contributed towards an efficient referral system by providing operational funds for the ambulance service in public facilities.

Additionally, the policy maker respondents were determined to increase the number of lower health centres undertaking deliveries. In this regard, there is an ongoing drive to equip every health centre II in the district of Gulu with a mini-theatre and recruit at least a midwife since these health centres tend to serve a large number of communities and are more accessible to community members.

## Discussion

Our study has demonstrated what lies behind the poor state of EmONC services in post-conflict Burundi and Northern Uganda, in the process moving away from the figures to the contextual factors and challenges that interact to engender poor delivery of EmONC services. We also highlight key strategies employed by the relevant stakeholders to improve the availability and delivery of quality EmONC services to the general population. While many studies in the past have focused on facility-based assessment on the status of EmONC services and barriers faced by women and communities to access quality EmONC services, this study focuses on EmONC supply stakeholders to understand why in spite of the strong evidence of the importance of access to and quality of EmONC services in reducing maternal and newborn morbidity and mortality the delivery of quality EmONC services remains poor in some settings. Additionally, with stronger global commitment to reducing child mortality and improving maternal health under the United Nations initiative of the Millennium Development Goals (MDGs) one would expect the delivery of quality EmONC services to be a priority in countries experiencing a high burden of maternal and neonatal morbidity and mortality. Our key message is that post-conflict health systems face different challenges in the delivery of quality EmONC services and as such any initiatives to improve the delivery of quality EmONC services in such settings must take into consideration the unique contextual challenges. A ‘one size fits all’ approach may fail to effectively address the problem.

### Current state of EmONC

The national availability of EmONC services appears to be a major public health concern in Burundi and Northern Uganda as suggested by our findings. Up-to-date information on the national availability and coverage of EmONC is difficult to find, even among the key EmONC supply stakeholders. A 2010 EmONC need assessment in Burundi puts the percentage of national availability of the recommended minimum EmOC services at 27% [38]. The corresponding available data for Uganda is for 2003 and stands at 34% [39]. At such low coverage rates, many women and newborns, especially in rural areas will remain out of reach for this important lifesaving medical interventions. The non-delivery of EmONC functions by officially designated EmONC facilities observed in our study corroborates the findings of an earlier countrywide study on availability of EmOC services in Uganda [40]. Kim et al. [41] found a similar situation in Afghanistan, where up to 42% of peripheral facilities that were expected to perform all 9 signal functions required for CEmONC did not provide such services. Additionally, a cross-sectional facility-based survey in Kenya revealed that majority of the facilities surveyed were not providing the designated EmOC services, and a huge equity gap in service provision existed between urban and rural areas in favour of urban areas [42], a similar observation in our study sites. It was therefore not surprising that many of our study participants reported that access to and quality of EmONC services was one of the most important health challenges facing their respective countries.

### Barriers to effective EmONC delivery

Our findings suggest that the effective delivery of quality EmONC services in post-conflict settings in Burundi and Northern Uganda is undermined by a lot of human resources-related, and systemic and institutional challenges. We demonstrate that while some of the challenges are similar across the study settings, others are unique to specific areas. With respect to the human resources-related challenges, we identified *seven* subthemes, with *four* of them common to both Burundi and Northern Uganda, and *three* common only to Northern Uganda. Concerning the systemic and institutional failures, we identified *nine* subthemes; *three* common to both study sites, *three* common to only Northern Uganda, and *three* common to only Burundi. These findings broadly suggest that EmONC supply stakeholders in Northern Uganda face more challenges in the delivery of quality EmONC services compared to their counterparts in Burundi. In this regard, we observed that the human resources-related challenges of domoralised personnel, chronic absenteeism in rural areas, and poor coordination among key EmONC personnel were only reported in Northern Uganda. Additionally, the systemic and institutional failures reported only for Burundi were focused on poor allocation of EmONC resources, and weak EmONC curriculum and training, while those reported only for Northern Uganda were focused on inefficient drug supply and general referral system. Arguably, one of the most important interventions that has alleviated the challenges in the delivery of EmONC in Burundi is the implementation of the performance-based financing programme in the country. Through the programme, health facilities are better supported with essential supplies, in addition to some financial incentives based on the quantity and qualitative of specific services administered. This has possibly improved morale and motivation among personnel.

The barriers in the delivery of quality EmONC services observed in our study are largely similar to those that have been reported across other conflict and post conflict settings. Previous global surveys among some key stakeholders have identified the following barriers: lack of funds; inadequate infrastructure; shortage of essential medicines, equipment and supplies; shortages of qualified staff; insufficient data collection; gaps in communication and emergency transport systems; high staff turnover; and lack of guidance in implementation among others [2,16]. Furthermore, eight years following the end of the conflict in Sierra Leone, Oyerinde at al. [43] observed that among facilities offering delivery services in the country, none was offering the complete designated BEmOC services and the available CEmOC facilities were poorly distributed, with a crowding of facilities in a few districts and a complete absence in many others. They equally found that effective EmOC delivery was hampered by severe shortages in personnel, equipment and supplies and an unreliable supply of utilities. In a four country study involving Kenya, Rwanda, Sudan and Uganda, Pearson and Shoo [44] identified shortage of trained staff, poor basic infrastructure such as lack of electricity and water supplies, inadequate supply of drugs and essential equipment, poor working conditions and staff morale, lack of communication and referral facilities among others as key barriers to providing 24-hour quality EmOC services especially in remote and rural areas. Furthermore, Ameh et al. [45] found that the lack of basic supplies, drugs, medical equipment and supportive policy remained key barriers to the non-use of new skills and knowledge acquired by EmOC personnel. These previous findings and those of our study suggest that while health systems recovering from armed conflicts may struggle to provide quality EmONC services, the underlying causes for this may differ from one setting to another. As such, implementing the same package of interventions to improve the delivery of EmONC services across different conflict and post-conflict settings may not be an effective strategy. Also, the findings of Ameh et al. [45] highlights the need for governments and their development partners to equally invest in both personnel training and provision of supplies if important improvements to efficient delivery of EmONC to the general population have to be achieved.

Unfortunately data and information on the trends of government expenditure on EmONC-related services and the strength of the EmONC workforce does not appear to be publicly available in Burundi and Northern Uganda. However, available data on the density of doctors, nurses and midwives per 10, 000 population for Burundi and Uganda stands at 2.2 (2004) and 14.2 (2005) respectively [33], far below the World Health Organization (WHO) recommended threshold of 23. Additionally, between 2000 and 2007, WHO estimated that the total number of physicians and midwives in Burundi and Uganda were 200 & 1,348, and 2,209 & 18,969 respectively [46]. A review of the membership of the Uganda Nurses and Midwives’, and Medical and Dental Practitioners’ Councils reveal a current membership of 27,812 and 4,746 respectively as of early February 2015 [47,48], giving a total of 32,558. With the population of Uganda estimated at 38.5 million [49], the recommended minimum number of health workers (doctors, nurses and midwives) should be 89,240. This implies that Uganda is experiencing a shortage of 56,682 health workers. Data from Burundi is hard to find and might be worse considering the very low density of doctors, nurses and midwives per 10,000 population compared to Uganda. Furthermore, Burundi and Uganda are not on course to meeting the UN MDG targets of reducing maternal and under-five mortality ratios [33]. With newborns accounting for 35% of the 43,000 under-five deaths recorded in Burundi in 2012 [33], there is an ever greater need to strengthen emergency neonatal care services in the country, especially for managing low birth weight and birth asphyxia [50]. In Uganda, neonatal care services have also been coming under criticism as a recent study revealed that majority of public health facilities lack basic equipment to resuscitate newborns, resulting in high newborn deaths [51]. These analyses reveal the depth of the problem of acute shortage of health workers in the study sites, and will require extraordinary measures for over many years for the problem to be addressed.

### Strategies to improve EmONC delivery

The current strategies employed by local EmONC supply stakeholders across the study sites to improve the delivery of EmONC services broadly reflected the challenges that they are experiencing. The strategies from Burundi were limited to capacity building of essential personnel and equipping of EmONC facilities, improving the ambulance service for emergency referrals, and harmonising and strengthening the curriculum and training for EmONC. On the other hand, those from Northern Uganda were more extensive and consisted of supporting the training of midwives, providing facilities with EmONC supplies, increasing the salaries of doctors and number of basic EmONC facilities in rural areas, strengthening the referral system, and better support for staff in rural areas. When compared to the challenges identified in each of the study sites, the respective current strategies are inadequate and do not go far enough to address all the challenges. This suggests that the problems with poor delivery of EmONC services may persist across the sites for some time.

A wide range of strategies have been proposed for improving the delivery of quality EmONC services in crisis and other low-resource settings with the goal of better maternal and newborn health outcomes. For example, the provision of EmOC training to essential personnel in post-conflict Somaliland saw a 100% provision of EmOC services by designated BEmOC and CEmOC facilities from a baseline of 43% and 56% respectively [45]. In Afghanistan, Turkmani et al. [52] have demonstrated that a comprehensive national midwifery education system involving an 18-month community midwifery education programme for community-based health facilities has improved rural women's access to skilled care at birth and subsequently reduced maternal deaths. To further address the barrier of acute shortage of human and material resources in conflict settings, Lee [53] has proposed a model of public-private partnership, where the local government and NGOs come together to better deliver maternal health care to the affected population. In a conflict-affected area in the Philippines, they showed how the local government provided NGOs space in government health facilities with the NGOs bringing in critical supplies, personnel and other supplies. These are service delivery models that can be explored within our study sites to address the persistent problem of shortage of essential EmONC personnel and medical supplies. An earlier study in Uganda found that the single most effective intervention to reduce maternal deaths was the availability of midwives at the level of the EmONC facility [40]. Additional studies have identified midwives as the backbone of any effective EmONC programme [52,54]. In spite of the well-acknowledged benefits of midwives to drive down maternal deaths at health facilities, a chronic shortage of midwives exists in our study sites. For example, in 2010 the Gulu district health officer identified a gap of 316 health workers especially for the rural areas where health centres have been constructed, but have not been operational [55]. The few personnel who were recruited tend to leave to the neighboring Sudan due to poor pay [55], similar concerns to what we observed in our study.

Furthermore, Wick and Hassan [56] have suggested better support, supervision and equipping of key EmONC personnel, especially midwives to be able to assist pregnant and birthing women and newborns at any time and in any circumstances. Kongnyuy et al. [57] have equally identified improvements in human resources, referral system, health infrastructure, health information system among others as important strategies to overcome the barriers to EmOC services in resource poor settings like Burundi and Uganda. While some of these are currently being implemented across Burundi and Northern Uganda, huge underlying challenges especially with respect to coverage remain as most of the major facilities tend to be located in urban centres while the majority of people still live in rural and semi-urban settings. There is therefore a need to extend the services to rural and semi-urban areas where the demand for such services is high. In that regard, Tayler-Smith et al. [58,59] have shown that a basic ambulance referral network coupled with the provision of quality EmOC is a feasible and cost effective intervention to substantially reduce maternal morbidity and mortality in rural Burundi. It should be highlight that even when EmONC resources are available, efficient coordination among key stakeholders and allocation of resources is equally important. In post-conflict settings such as Nepal where substantial improvements in maternal health have been observed, this has partially been associated to strong international commitment and support of Nepal’s health system during and after the conflict, and better coordination among key stakeholders involved in the provision of health services [60].

The availability and provision of quality EmONC services remain the most effective way of reducing maternal and newborn deaths and disabilities [40,61–63]. The relatively high maternal and neonatal mortality ratios in our study sites may very much reflect the challenges affecting the effective delivery of such services. The AMDD recommends that any EmONC services must be supported among others by evidence-based policies [14]. Taking this into consideration, there is a need for key EmONC stakeholders in our study sites to explore our study findings to improve the delivery of quality EmONC services. While most attention in the past has been focused on effective referral, provision of essential equipment, supplies and medication, and recruitment of qualified staff, our study suggests that more attention should also be paid to the issues around staff motivation, supervision, support and recognition; burn-out and turnover; and improved living and working conditions, all supported by our findings in Northern Uganda. For Burundi, the development of a strong and nationwide EmONC curriculum coupled with better allocation of EmONC resources seems to be important focus areas. Across the sites, effective and reliable data collection and monitoring systems need to be instituted and strengthened while also ensuring that EmONC facilities are equitably distributed.

**Fig 1:**
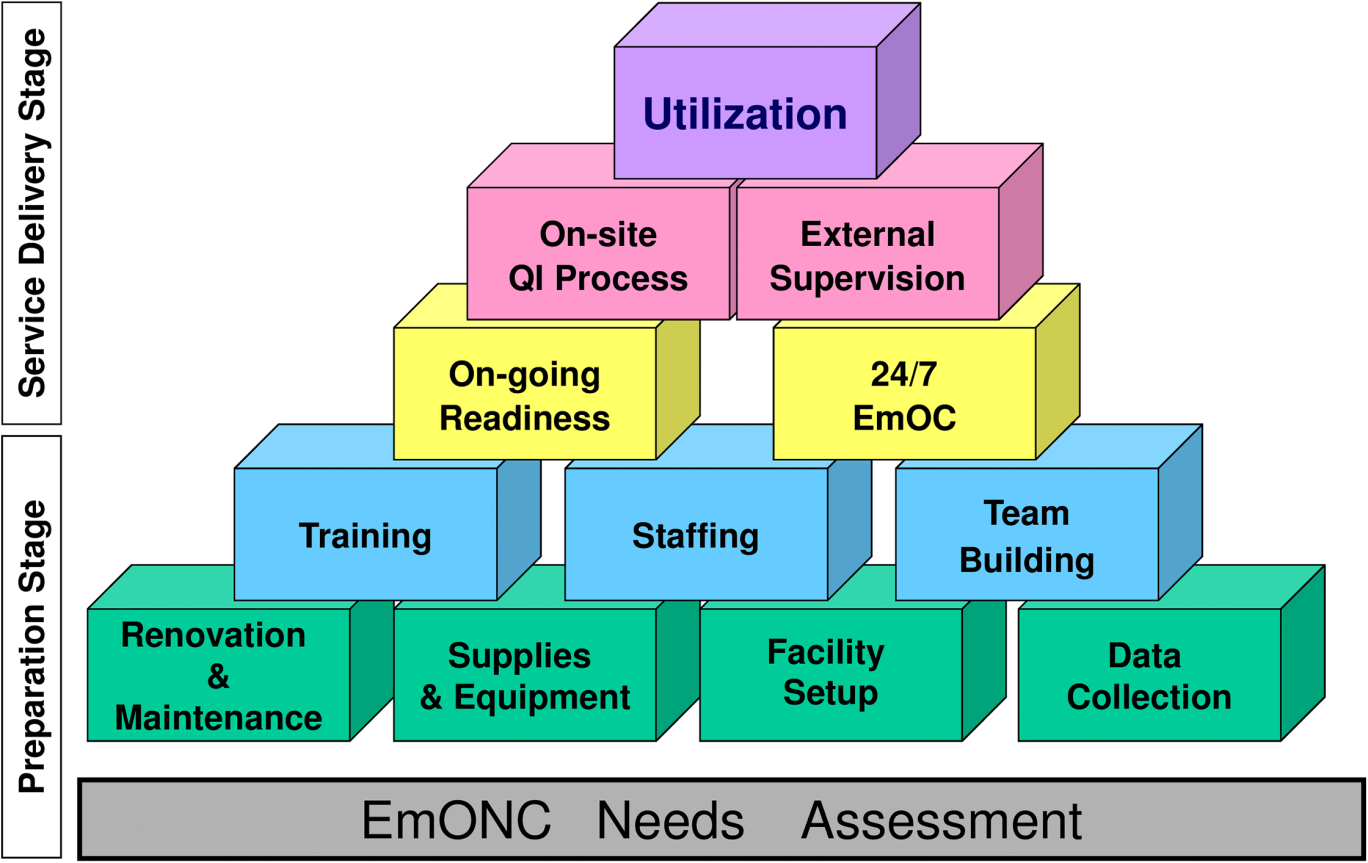
AMDD EmOC Building Blocks Framework (Source: AMDD). Reprinted from “Averting Maternal Death and Disability Program Report: 1999–2005. Averting Maternal Death and Disability program, Mailman School of Public Health, Columbia University. October 2006. Available at: http://www.amddprogram.org/v1/resources/1999_2005_report.pdf” under a CC BY license, with permission from “Averting Maternal Death and Disability program at Columbia University Mailman School of Public Health (www.amddprogram.org)”, original copyright 2005. Permission to reprint is provided by Lynn Freedman, Director of AMDD.

Our findings suggest that the current strategies being undertaken at the various study sites to improve the delivery of EmONC services are not far-reaching enough. There is a need to invest more resources in addressing the root causes of shortage of qualified personnel, demoralised and demotivated staff, high turnover, and staff absenteeism in rural areas among others in Northern Uganda. This will involve more focus on personnel-centred interventions geared towards improving their living and working conditions, in addition to broader health system interventions. The current performance-based financing programme in Burundi [64] is a unique opportunity for the authorities to substantially invest in broader EmONC support and strengthening to stem the current high maternal and newborn mortality in the country as highlighted in the recent Countdown report [33]. The AMDD programme EmOC Building Blocks framework (Fig 1, permission for reprint at S1 File) provides a useful model for improving the delivery of EmONC services from the preparatory to the service delivery stage. Using this model the Reproductive Health Response in Crises (RHRC) Consortium increased the availability of EmOC services 24 hours a day and 7 days a week in 12 conflict-affected project settings in 9 countries, in the process implementing interventions such as construction of health facilities, supply of equipment and supplies, placement of qualified staff and onsite technical assistance [17]. This is useful model that can be explored by key EmONC stakeholders in Burundi and Northern Uganda to step-up the delivery of quality EmONC services, with the goal of making access to quality EmONC services a reality for all women and newborns.

## Conclusions

Our study has highlighted that concerns around the availability and quality of EmONC services remain one of the most critical challenges facing maternal and neonatal health in post-conflict Burundi and Northern Uganda. The effective delivery of EmONC services have been compounded by a number of human resources-related and systemic and institutional challenges, with some common across the sites while others are unique to each site. The main common barriers include an acute shortage of trained personnel, medical supplies and equipment; high burn-out and turnover; inequity in the distribution of EmONC facilities; and poor data collection and monitoring and surveillance system. Barriers unique only to Burundi comprise of poor harmonisation and quality of EmONC training programmes; and poor allocation of EmONC resources. Finally, the main barriers unique to Northern Uganda include: inefficient referral and drug supply systems; high levels of staff absenteeism in rural areas; and poor coordination among key EmONC personnel resulting in delays to provide emergency services. While a number of initiatives and interventions are currently employed to improve on the situation, more effective and well-coordinated strategies, especially within the health system are required to substantially improve the delivery of EmONC services in Burundi and Northern Uganda.

## Supporting Information

S1 File Permission to reprint the AMDD Model. (PDF)
