# Supplementary material for: Barriers in the Delivery of Emergency Obstetric and Neonatal Care in Post-Conflict Africa: Qualitative Case Studies of Burundi and Northern Uganda
Source: PLoS One. 2015 Sep 25;10(9):e0139120. doi: 10.1371/journal.pone.0139120 (PMC4583460; doi:10.1371/journal.pone.0139120)
Supplement: S1 File — (PDF) [file pone.0139120.s001.pdf]

Primus Che Chi

From: Freedman, Lynn P. <lpf1@cumc.columbia.edu>
Sent: 9. februar 2015 21:50
To: Primus Che Chi
Subject: Re: AMDD EmOC Building blocks
Attachments: Gill et al Walk-through - AMDD bldg blocks IJGO 2005.pdf; AMDD EmONC Building Blocks rev 2006[1].ppt

Dear Primus,
I remain unsure which publication had the exact version of the EmOC building blocks that you sent? I attach the original version that was published in the IJGO paper by Gill et al. I also attach a powerpoint slide of the updated one that we now use, which may be closer to the one you sent.
You have our permission to reproduce the building blocks in the ppt (with citation to Averting Maternal Death and Disability program at Columbia University Mailman School of Public Health (www.amddprogram.org)). This is the AMDD revision of the AMDD building blocks in Gill et al. I am not certain if PLOS One would require you to obtain permission from IJGO directly.

Please send us your paper when it is published. I'm sure that our team will be very interested to read it, if it uses the building blocks.

Best,
Lynn

From: Primus Che Chi <prichi@prio.no>
Date: Saturday, February 7, 2015 at 8:55 AM
To: "Freedman, Lynn P." <lpf1@cumc.columbia.edu>
Subject: RE: AMDD EmOC Building blocks

Dear Lynn,
Thanks for the very quick response. I think the best option for me will be to get a similar version from AMDD rather than modifications from other authors. I will therefore be delighted if you can provide me the version from AMDD.

Thanks,
Primus

From: Freedman, Lynn P. [mailto:lpf1@cumc.columbia.edu]
Sent: 7. februar 2015 14:50
To: Primus Che Chi
Subject: Re: AMDD EmOC Building blocks

Dear Primus,
We are pleased to give the permission of AMDD to publish the framework with proper attribution to AMDD, but I am not sure where this one was previously published and whether that journal has different requirements for copyright permission. In other words, you may be required to obtain the permission from the journal that published the version you are using if it is the journal (rather than the authors) that holds the copyright. Which paper are you referring to?

Sorry for these complications. We are very happy for other researchers to use this image. The legal issue of copyright permission might depend on the journal where this particular figure was first published.

Best,
Lynn

From: Primus Che Chi <prichi@prio.no>
Date: Saturday, February 7, 2015 at 7:17 AM

To: "Freedman, Lynn P." <lpf1@cumc.columbia.edu>
Cc: Marta Schaaf <mls2014@cumc.columbia.edu>
Subject: RE: AMDD EmOC Building blocks

Dear Lynn,
Thank you for getting back in touch. The version of the AMDD EmOC Building Blocks Framework has been published elsewhere. Kindly find attached the figure we plan to use for our PLOS One article.

Thanks again for your kind understanding.

Best regards,
Primus

From: Freedman, Lynn P. [mailto:lpf1@cumc.columbia.edu]
Sent: 6. februar 2015 21:08
To: Primus Che Chi
Cc: Schaaf, Marta L.
Subject: AMDD EmOC Building blocks

Dear Primus,

Thank you for your inquiry. Is it possible for you to send me the version of the AMDD EmOC Building Blocks Framework you would like to use in the PLoS One paper. There were several versions over time, so I want to make sure that we are talking about the same version. Is it a version that you found published elsewhere?

Thank you and best regards,
Lynn Freedman

Lynn P. Freedman, JD, MPH
Director, Averting Maternal Death and Disability Program (AMDD)
Professor of Population and Family Health at CUMC
Mailman School of Public Health, Columbia University
60 Haven Avenue, B-3
New York, NY 10032
USA

Tel: +1 212 304 5281
LPF1@columbia.edu

---------- Forwarded message ----------
From: **AMDD** <info@amddprogram.org>
Date: Thu, Jan 29, 2015 at 7:20 AM
Subject: Form submission from: Contact Form
To: peb2001@gmail.com

Submitted on Thursday, January 29, 2015 - 07:20
Submitted by anonymous user: [82.199.16.14]
Submitted values are:

Name: Primus Che Chi
Email Address: prichi@prio.org
Message:
Dear Sir/ madam,

Hope my mail finds you well. I am Primus Che, a doctoral researcher at the Peace Research Institute Oslo (PRIO) and PhD candidate at the Faculty of Medicine, University of Oslo. I request permission for the open-access journal PLOS ONE to publish the "AMDD EmOC Building Blocks Framework " under the Creative Commons Attribution License (CCAL) CC BY 3.0 (http://creativecommons.org/licenses/by/3.0/us/). Please be aware that this license allows unrestricted use and distribution, even commercially, by third parties. Please reply and provide explicit written permission to publish the "AMDD EmOC Building Blocks Framework "under a CC BY license.

Thanks and best regards,
Primus

The results of this submission may be viewed at:
http://www.amddprogram.org/node/40/submission/3709

--

Zoë Stopak-Behr
Research Assistant
AMDD, Columbia University
info@amddprogram.org

Website: http://www.amddprogram.org/
Twitter: @AMDD_Program
